# Induction of apoptosis by the ADEPT agent ZD2767: comparison with the classical nitrogen mustard chlorambucil and a monofunctional ZD2767 analogue

**DOI:** 10.1054/bjoc.2001.1947

**Published:** 2001-09

**Authors:** N R Monks, D C Blakey, N J Curtin, S J East, A Heuze, D R Newell

**Affiliations:** Cancer Research Unit, University of Newcastle upon Tyne, Newcastle upon Tyne, NE2 4HH, Tyne and Wear; Cancer and Infection Bioscience Department, AstraZeneca Pharmaceuticals, Alderley Park, Macclesfield, Cheshire, SK10 4TG, UK

**Keywords:** apoptosis, ZD2767, chlorambucil, monofunctional ZD2767 analogue, colorectal tumours

## Abstract

ZD2767P is a phenol mustard glutamate prodrug which is currently being developed for Antibody Directed Enzyme Prodrug Therapy (ADEPT). In ZD2767 ADEPT an active bi-functional alkylating drug, ZD2767D (4-[N,N-bis(2-iodoethyl)amino]phenol), is generated following cleavage of ZD2767P by the bacterial enzyme carboxypeptidase G2 (CPG2) which is targeted to the tumour by conjugation to the F(ab′)_2_ fragment of the anti-CEA antibody A5B7. The aim of the studies described here was to identify the mode of cell death induced by ZD2767P + CPG2 in comparison to the established nitrogen mustard chlorambucil. The contribution of bifunctional and monofunctional ZD2767 DNA lesions to cell death induction was investigated using a monofunctional ZD2767D analogue. Apoptosis in LoVo tumour cells was studied by three different methods (nuclear morphology, annexin V staining and TUNEL). Levels of apoptosis detected using the three assays were similar, and each drug treatment produced apoptosis at levels above those in control cells at concentrations which resulted in tumour cell growth inhibition. The bi-functional compounds, ZD2767P + CPG2 and chlorambucil, induced apoptosis in a concentration and time dependent manner, with equitoxic concentrations producing equivalent levels of apoptosis. In contrast, the mono-functional ZD2767D analogue at 100 μM resulted in the maximal level of apoptosis at 25 h with no further increase over the following 72 h. These studies have demonstrated that apoptosis is the mechanism of cell death induced by the ZD2767 ADEPT system, and that levels of apoptosis produced by ZD2767 are similar to those observed at equitoxic concentrations of the classical nitrogen mustard chlorambucil. The mono-functional ZD2767 analogue also induced apoptosis, but with a different time course and characteristics. In conjunction with previous data, these studies have shown that the potent activity of ZD2767 can be attributed to the ability of the compound to induce bifunctional DNA lesions and engage apoptosis. © 2001 Cancer Research Campaign http://www.bjcancer.com


*British Journal of Cancer* (2001) **85**(5), 764–771
© 2001 Cancer Research Campaign
doi: 10.1054/ bjoc.2001.1947, available online at http://www.idealibrary.com on IDEAL

http://www.bjcancer.com

### Induction of apoptosis by the ADEPT agent ZD2767: comparison with the classical nitrogen mustard chlorambucil and a monofunctional ZD2767 analogue

**NR Monks[1], DC Blakey[2], NJ Curtin[1], SJ East[2], A Heuze[2] and DR Newell[1]**

[1]Cancer Research Unit, University of Newcastle upon Tyne, Newcastle upon Tyne, Tyne and Wear NE2 4HH; [2]Cancer and Infection Bioscience Department, AstraZeneca Pharmaceuticals, Alderley Park, Macclesfield, Cheshire SK10 4TG, UK

**Summary** ZD2767P is a phenol mustard glutamate prodrug which is currently being developed for Antibody Directed Enzyme Prodrug Therapy (ADEPT). In ZD2767 ADEPT an active bi-functional alkylating drug, ZD2767D (4-[N,N-bis(2-iodoethyl)amino]phenol), is generated following cleavage of ZD2767P by the bacterial enzyme carboxypeptidase G2 (CPG2) which is targeted to the tumour by conjugation to the F(ab′)$_2$ fragment of the anti-CEA antibody A5B7. The aim of the studies described here was to identify the mode of cell death induced by ZD2767P + CPG2 in comparison to the established nitrogen mustard chlorambucil. The contribution of bifunctional and monofunctional ZD2767 DNA lesions to cell death induction was investigated using a monofunctional ZD2767D analogue. Apoptosis in LoVo tumour cells was studied by three different methods (nuclear morphology, annexin V staining and TUNEL). Levels of apoptosis detected using the three assays were similar, and each drug treatment produced apoptosis at levels above those in control cells at concentrations which resulted in tumour cell growth inhibition. The bi-functional compounds, ZD2767P + CPG2 and chlorambucil, induced apoptosis in a concentration and time dependent manner, with equitoxic concentrations producing equivalent levels of apoptosis. In contrast, the mono-functional ZD2767D analogue at 100 μM resulted in the maximal level of apoptosis at 25 h with no further increase over the following 72 h. These studies have demonstrated that apoptosis is the mechanism of cell death induced by the ZD2767 ADEPT system, and that levels of apoptosis produced by ZD2767 are similar to those observed at equitoxic concentrations of the classical nitrogen mustard chlorambucil. The mono-functional ZD2767 analogue also induced apoptosis, but with a different time course and characteristics. In conjunction with previous data, these studies have shown that the potent activity of ZD2767 can be attributed to the ability of the compound to induce bifunctional DNA lesions and engage apoptosis. © 2001 Cancer Research Campaign http://www.bjcancer.com

**Keywords**: apoptosis; ZD2767; chlorambucil; monofunctional ZD2767 analogue; colorectal tumours

Attempts to improve the selectivity of anti-cancer chemotherapy have led to the development of treatments which target anti-tumour agents to the tumour. One of the most recent developments in targeted therapy is Antibody Directed Enzyme Prodrug Therapy (ADEPT) (Bagshawe, 1987). ADEPT utilizes a monoclonal antibody raised against a tumour-associated antigen, linked to an enzyme, which cleaves a relatively inactive prodrug into a highly diffusable cytotoxic species within the tumour, killing both antigen positive and negative neoplastic cells.

ZD2767 is the latest ADEPT system to be developed and has recently completed a Phase 1 clinical evaluation in patients with carcinoembryonic antigen (CEA)-expressing tumours. The ZD2767 ADEPT system utilizes the A5B7 F(ab′)$_2$-carboxypeptidase G2 (CPG2) conjugate (ZD2767C) which is targeted at CEA expressing tumours. The enzyme specifically activates a di-iodophenol mustard glutamate prodrug (4-[N,N-bis(2-iodoethyl)amino]phenoxy-carbonyl L-glutamic acid, ZD2767P, Figure 1A) to the potent di-iodophenol mustard (4-[N,N-bis(2-iodoethyl)amino]phenol, ZD2767D, Figure 1B), via the cleavage of the carbamate bond releasing the glutamate moiety (Springer et al, 1995; Blakey et al, 1996).

Previous investigations have demonstrated that ZD2767P + CPG2 is both cytotoxic and growth inhibitory in human colorectal and NSCLC tumour cell lines (Monks et al, submitted). Comparisons of ZD2767P + CPG2 with the classical aniline mustard chlorambucil have identified similar patterns of activity in both types of tumour cell lines; however, ZD2767P + CPG2 was at least 100-fold more potent than chlorambucil. ZD2767P + CPG2 rapidly forms DNA-DNA interstrand cross-links in a concentration-dependent manner, the lesion thought to be responsible for the cytotoxicity of bifunctional agents such as chlorambucil, melphalan and HN2.

The mechanism underlying ZD2767P + CPG2-induced cell death has not been determined, although it has been stated that ‘almost without exception toxins induce death by apoptosis’ (Hickman and Boyle, 1997). The structurally and chemically related aniline mustard chlorambucil has been reported to induce apoptosis in B-cell chronic lymphocytic leukaemia (B-CLL) cells both in vivo (Begleiter et al, 1994) and in vitro (Pepper et al, 1997), in a concentration-dependent manner. Studies in HL-60 human promyelocytic leukaemia cells also demonstrated that chlorambucil (3 mM) induces apoptosis; however, increasing the concentration to 30 mM produced cell death by necrosis (Lennon et al, 1991).

*Received 24 January 2001*
*Revised 1 June 2001*
*Accepted 4 June 2001*

*Correspondence to:* DR Newell

BJOC 01-1947 764-771 20/8/01 3:14 pm Page 765

The investigations presented here were undertaken to determine the mode of cell death induced by treatment with ZD2767P + CPG2. LoVo colorectal tumour cells were chosen for these studies as they are sensitive to, and have been extensively used in in vitro and in vivo studies with, the ZD2767 ADEPT system (Blakey et al, 1996; Springer et al, 1995). Apoptosis was determined using 3 distinct methods which assess apoptosis-related morphological changes. Firstly, apoptotic nuclear morphology was assessed by staining cells with Hoechst 33258, a bis(benzimidazole) dye which becomes highly fluorescent when bound to A-T rich sequences of double-stranded DNA allowing the assessment of chromatin structure by fluorescent microscopy (Lootiens et al, 1990). In addition, two flow cytometric assays were also used to measure apoptosis, each directed at different phenomena which arise during apoptotic cell death. The annexin V-propidium iodide dual staining assay (Vermes et al, 1995) was used to detect the loss of plasma membrane phospholipid asymmetry, which results in phosphatidylserine being exposed on the surface of the cell. Loss of membrane asymmetry occurs early in apoptosis (Fadok et al, 1992) and precedes the loss of membrane selective permeability (detected by the exclusion of PI) and other events normally associated with apoptosis such as chromatin condensation (Martin et al, 1995). The second flow cytometric method used was the terminal deoxynucleotidyl transferase-mediated dUTP nick end labelling (TUNEL) assay which measures the extent of DNA fragmentation, an event which occurs once specific DNA endonucleases have been activated (Gavrieli et al, 1992; Sgonc et al, 1994).

The aim of the studies described here was to determine the mechanism, time course and level of cell death induced by ZD2767P + CPG2 in LoVo colorectal tumour cells as assessed by three distinct morphological features of apoptosis. In addition, the importance of mono- and bi-functional lesions to drug-induced cell death was studied by comparing ZD2767P + CPG2-induced apoptosis with that induced by the bifunctional aniline mustard chlorambucil (Figure 1D) and a structurally related monofunctional ZD2767D analogue (Figure 1C). With a targeted therapy such as ADEPT, it could be argued that the mechanism of cell killing is not relevant to the ultimate anti-tumour effect, provided the tumour cell is effectively targeted and killed. However, there is increasing evidence that an inability to engage apoptosis can represent a resistance mechanism to cytotoxic drugs, and many of the signal transduction pathways required for apoptotic cell death have been defined (Hickman and Boyle, 1997). Conversely, local inflammatory responses could be an important determinant of tumour response following cell death by necrosis. Hence an understanding of the mechanism of cell killing following ADEPT therapy could ultimately be of value in selecting tumours and patients who are more likely to respond to treatment by virtue of the presence of factors required for the relevant mechanism of cell death.

### MATERIALS AND METHODS

### Materials

The prodrug ZD2767P was synthesized as described previously (Springer et al, 1995), as was the monofuntional ZD2767D analogue (Monks et al, submitted). Chlorambucil was purchased from Sigma Chemical Co. (Poole, Dorset, UK) and CPG2 was provided by CAMR (Salisbury, Wiltshire, UK). The LoVo colorectal tumour cell line was obtained from the European Collection of Animal Cell Cultures (ECACC, Wiltshire, UK). All

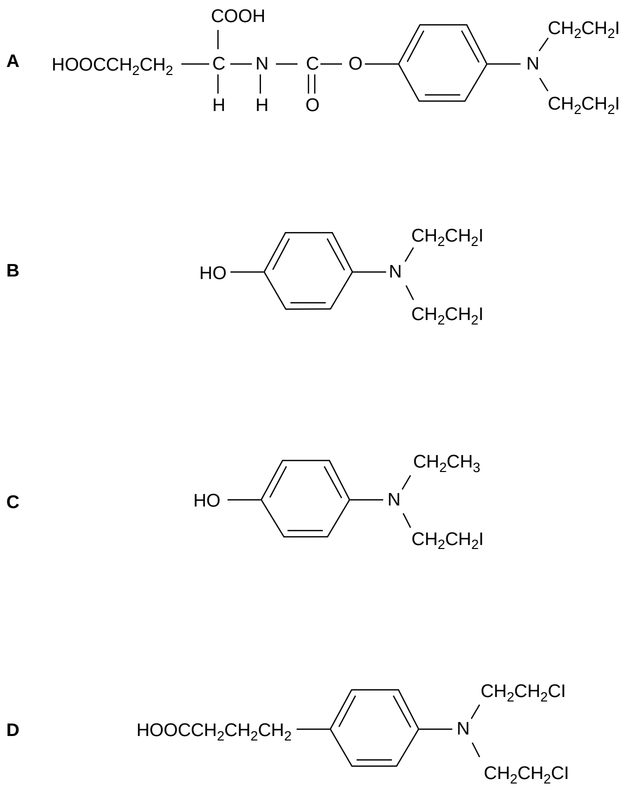

**Figure 1** Structure of the compounds investigated. (**A**) 4-[*N*,*N*-bis (2-iodoethyl)amino]phenoxycarbonyl L-glutamic acid prodrug (ZD2767P), (**B**) 4-[*N*,*N*-bis(2-iodoethyl)amino]phenol drug (ZD2767D), (**C**) 4-[*N*-(iodoethyl)-*N*-ethyl-amino]phenol drug (monofunctional ZD2767D) and (**D**) 4-[p-bis(2-chloroethyl)aminophenyl]butyric acid (chlorambucil)

cell lines were maintained in RPMI 1640 (complete) medium containing 2 mM L-glutamine (Gibco-BRL Life Technologies Ltd, Paisley, UK), supplemented with 10% (v/v) FCS (Globerpharm Ltd, Esher, Surrey, UK) and incubated at 37˚C, 5% $CO_2$. The LoVo cells were routinely tested to exclude mycoplasma infection (Chen, 1977). All other reagents were supplied by Sigma Chemical Co., (Poole, Dorset, UK), unless stated otherwise.

### Drug treatment and cell growth inhibition

On day 0, exponential growing LoVo colorectal tumour cells were harvested, and $2 \times 10^5$ cells seeded in 10 ml of medium. On day 3, the medium was removed from each flask and replaced with fresh medium, with the exception of cells treated with ZD2767P which received medium containing 1 unit/ml of CPG2. Each compound was prepared from solid immediately prior to each experiment, by serial dilution in DMSO to 100 × the final required concentration. The cells were exposed to each drug for 1 h, with control cells being exposed to a similar concentration of DMSO alone (1% v/v). Following the drug exposure, the medium was removed and replaced with 10 ml of fresh drug-free medium. One flask treated with each drug concentration and a control flask were taken 1 h after the end of the drug exposure, and at 24-hour intervals thereafter, and the level of apoptosis assessed using the three assays. All three drug treatments (ZD2767P + CPG2, chlorambucil and the monofunctional ZD2767D analogue) were studied concurrently in each experiment in order to allow the direct comparison of results.

*© 2001 Cancer Research Campaign*

BJOC 01-1947 764-771 20/8/01 3:14 pm Page 766

To determine drug-induced growth inhibition, cells were seeded at a density of $2 \times 10^4$ cells/ml on day 0. On day 3 the medium was removed and replaced with of fresh medium, with the exception of the ZD2767P + CPG2-treated cells where the medium contained 1 unit/ml of CPG2. One unit of CPG2 is defined as the amount of enzyme required to hydrolyse 1 μmol of methotrexate per min per ml of reaction mixture at 37˚C (Sherwood et al, 1985). Each drug concentration (100 × final concentration in DMSO) was added to the cells, resulting in the same concentrations as those used in the apoptosis studies. The cells were exposed to each compound for 1 h, after which the medium was removed and replaced with fresh drug-free medium. Both the adherent cells and those in suspension were pooled and the cell number was measured at 1, 25, 49, 73 and 97 hours using a model Z1 Coulter Counter (Coulter Electronics, Bedfordshire, UK).

### Measurement of apoptosis by Hoechst 33258 nuclear morphology staining

After drug exposure and post-exposure incubation for the time indicated, the medium in which the cells were being cultured was removed and put aside. The cells were harvested using trypsin (Gibco-BRL Life Technologies Ltd) in Dulbecco's PBS containing 0.054 mM EDTA. Once released from the tissue culture plastic, the cells were returned to the original medium in which they had been cultured, and disagregated by pipetteing the suspension up and down 6–7 times in a 5 ml pipette, following which they were centrifuged at $300 \times g$ for 5 min. The supernatant was removed and the cell pellet re-suspended in 10 ml of ice cold PBS. One ml of each sample was transferred to an eppendorf tube, and then centrifuged at $300 \times g$ for 5 min at room temperature, following which the supernatant was removed and the cells fixed by resuspending the pellet in 20 μl of Carnoy's fixative (methanol:glacial acetic acid, 3:1 v/v) for 1 minute. After fixation, 20 μl of Hoechst 33258 (8 μg/ml in Dulbecco's PBS) was added to the cells and samples incubated in the dark for 5 min. The samples were subsequently mixed and 20 μl placed onto a glass slide under a cover slip. A fluorescent microscope (Zeiss, Heidleberg, Germany) fitted with an HBO 50 W high pressure mercury lamp (Osram, Germany) was used to count approximately 600 nuclei/slide, from which the percentage of apoptotic nuclei (condensed chromatin) was calculated.

### Measurement of apoptosis by Annexin V – Propidium iodide dual staining

LoVo colorectal cells were treated and harvested in ice cold PBS as described above. Each sample was centrifuged again at $300 \times g$ for 5 min, following which the supernatant was removed and the cells re-suspended at a density of approximately $1 \times 10^6$ cells/ml in binding buffer (HEPES buffered saline solution supplemented with 2.5 mM $CaCl_2$). 100 μl of cell suspension (approximately $1 \times 10^5$ cells) was then placed in an eppendorf tube to which 10 μl of FITC-labelled annexin V (AnV) (10 μg/ml) and 10 μl of propidium iodide (PI) (50 μg/ml) were added and mixed gently. The following four control samples were also produced in order to calibrate the flow cytometer: untreated control cells without AnV or PI stains, untreated control cells with only AnV stain; untreated control cells with only PI stain, and untreated control cells with both AnV and PI stains. The samples were then incubated for 15 min at room temperature in the dark, after which an additional 400 μl of binding buffer was added to each sample. The level of apoptosis was analysed using a FACScan flow cytometer, (Becton Dickinson, Oxford, UK) within 1 h of staining.

The flow cytometric analysis was performed according to manufacturer's instructions (Apoptosis Detection Kit, R&D Systems, Abingdon, UK). Accurate analysis of annexin V-PI labeled cells requires electronic compensation to exclude any overlap between the FITC and PI emission spectra. Compensation is achieved by setting the instrument using singly-stained control samples at the beginning of each analysis to adjust for any spectral overlap. Apoptotic cells were defined as those that were AV +ve/PI–ve, cells which did not stain for either AnV or PI were considered to be viable and non-apoptotic, and late apoptotic/ necrotic cells were those which were AnV +ve and PI +ve.

### Measurement of apoptosis by terminal deoxynucleotidyl transferase-mediated dUTP nick end labelling (TUNEL)

Following treatment, cells were harvested as described above and resuspended in ice cold PBS at a density of approximately $1 \times 10^7$ cells/ml. 100 μl of the cell suspension (≈ $1 \times 10^6$ cells) was transferred to an eppendorf tube and fixed for 30 min in 100 μl paraformaldehyde (4% w/v in PBS, pH 7.4, prepared prior to each assay) with regular mixing to avoid cell clumping. After fixation the cells were washed in 1 ml of ice cold PBS and centrifuged at $300 \times g$ for 8 min at room temperature. The supernatant was subsequently removed and the pellet re-suspended in 100 μl permeabilizing solution (0.1% (w/v) Triton® X-100, in 0.1% (w/v) sodium citrate in de-ionized water) and kept on ice for 2 min. The samples were then washed in 1 ml of ice cold PBS followed by centrifugation at $300 \times g$ for 8 min at room temperature, after which 35 μl of TUNEL reaction mixture (enzyme solution; terminal deoxynucleotidyl transferase from calf thymus, plus label solution; nucleotide mixture in reaction buffer containing FITC-dUTP. In Situ Cell Death Detection Kit, Boehringer Mannheim, Lewes, UK) was added to each sample. Prior to mixing the label solution with the enzyme, 100 μl of label solution was set aside to use as a control in the calibration of the flow cytometer. Three controls were used routinely during the assay, a blank (untreated cells in PBS only), a label control (untreated cells with only label solution) and a negative control (untreated cells with the TUNEL reaction mixture). A positive control, untreated cells incubated with 100 μl of DNAase 1 solution (100 μg/ml DNAase 1 in 30 mM Trizma base, 4 mM $MgCl_2$, 0.1 mM dithiothreitol, pH 7.2) for 10 min at room temperature, followed by treatment with TUNEL reaction mixture was also used.

The samples were incubated with the TUNEL reaction mixture for 1 h 37˚C in a humidified atmosphere, after which they were washed with 1 ml of ice cold PBS and centrifuged at $300 \times g$ for 8 min at room temperature. The cell pellet was then re-suspended in 400 μl of PBS, and fluorescence analysed using a FACScan flow cytometer (Becton Dickinson, Oxford, UK). Analysis of TUNEL stained samples involves comparison with an untreated control. The negative control cell population were gated at the upper end of the histogram, with approximately 1–5% of the cells being present within the gate, and this fraction of cells was considered apoptotic. Treated samples were then analysed and the same gate was applied, and any increase in the level of DNA breaks was detected as an increase in FITC-dUTP incorporated into the DNA, and visualized as an increase in the percentage of cells in the gated region.

© 2001 Cancer Research Campaign

### RESULTS

### Growth inhibition studies

To establish the relationship between growth inhibition and apoptosis, LoVo cell number was determined at that same drug concentrations and time points (1, 25, 49, 73 and 97 h) that apoptosis was investigated (Figure 2). ZD2767P + CPG2 caused a concentration-dependent decrease in the growth of LoVo colorectal tumour cells, with 1 and 10 μM producing cytostasis whereas 0.01 and 0.1 μM produced only limited growth inhibition. In the case of chlorambucil only the 1000 μM concentration resulted in complete growth inhibition. Slight growth inhibition was observed following exposure to 100 μM, whilst 1 and 10 μM chlorambucil resulted in growth similar to that of the control cells. Following exposure to the monofunctional ZD2767D analogue, complete growth inhibition was seen with cells treated with 100 and 1000 μM, whereas cell growth was not inhibited by either 1 or 10 μM monofunctional ZD2767D analogue. In previous studies (Monks et al, submitted) using the same assay conditions, the GI50 values for ZD2767P + CPG2, chlorambucil and monofunctional ZD2767D were 0.41 ± 0.14 μM, 79 ± 5 μM and 18 ± 5 μM, respectively, values consistent with the results presented here.

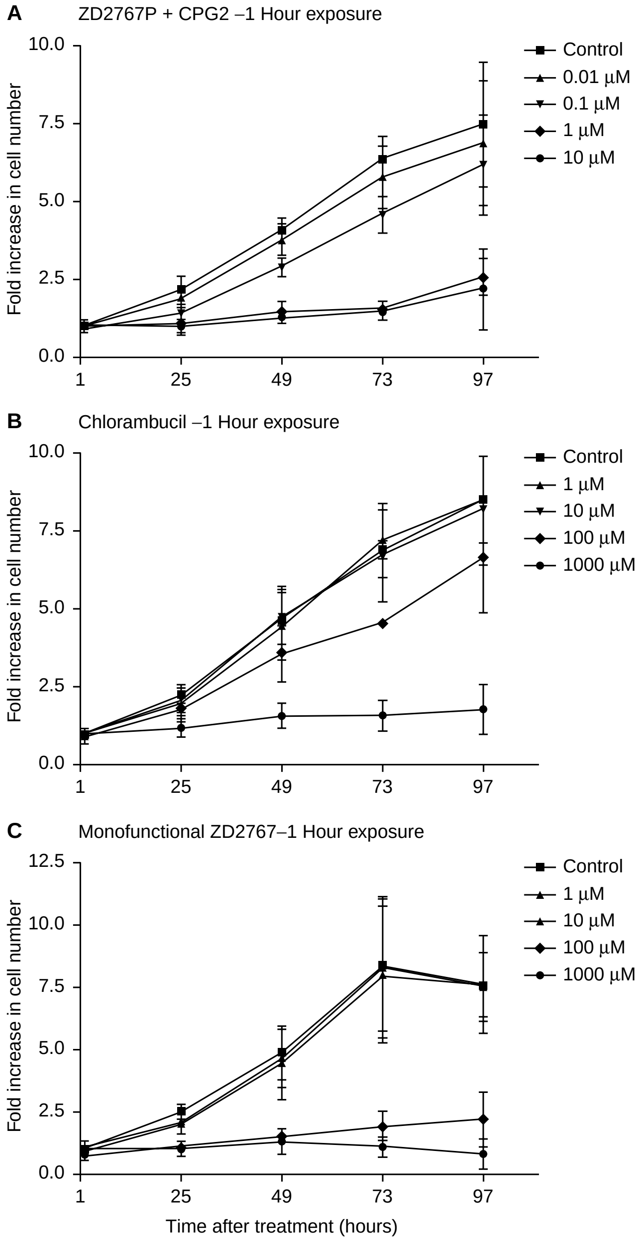

**Figure 2** Growth inhibition of LoVo colorectal tumour cells treated with ZD2767P + CPG2, chlorambucil or monofunctional ZD2767 analogue. Total cell number was determined after treatment with (**A**) ZD2767P + 1 unit/ml CPG2, (**B**) chlorambucil and (**C**) mono-functional ZD2767 analogue. The results are expressed as the fold increase in cell number over the 1 h control cell number. Each point represents the mean ± s.d. of $n = 3$ experiments performed as described in Materials and Methods

### Induction of apoptosis by ZD2767P + CPG2

To determine the mode, time course and concentration dependency of cell death induced by ZD2767P + CPG2, LoVo colorectal tumour cells were treated with four concentrations of ZD2767P + CPG2. LoVo cells were exposed to ZD2767P + CPG2 for 1 h prior to analysis of the levels of apoptosis 1, 25, 49, 73, and 97 hours after the exposure. Cell death detected by the three methods was concordant with respect to both the level and time course of apoptosis (Figure 3). In all three assays ZD2767P + CPG2-induced apoptosis was only seen following treatment with 1 and 10 μM drug; concentrations that produced cell growth inhibition (Figure 2). The levels of apoptosis detected at 0.01 and 0.1 μM did not differ from control levels as assessed by any of the three assays over the 97-hour experimental period.

Regardless of the assay used, drug-induced apoptosis was not detected 1 h after the end of the drug exposure. However, levels of apoptosis induced by both the 1 and 10 μM ZD2767P + CPG2 concentrations were above those seen in the control cells by 25 h and increased thereafter with time.

Overall, these data demonstrate that cytostasis produced by ZD2767P + CPG2 is associated with the induction of apoptosis which is detectable 25 h after the end of the 1-hour drug exposure, and increases thereafter with time. The use of three independent methods of measuring apoptosis, which all gave similar results, allows confidence to be placed in the results obtained.

### Induction of apoptosis by chlorambucil

To investigate whether the induction of apoptosis by ZD2767P + CPG2 is similar to that produced by a classical aniline mustard, LoVo colorectal tumour cells were exposed for 1 h to increasing concentrations of chlorambucil. As with ZD2767P + CPG2, chlorambucil produced apoptosis in a concentration-dependent manner (Figure 4). Again the data from the three apoptosis assays were in agreement, and demonstrated that levels of apoptosis were only greater than in control cells following treatment with 1000 μM chlorambucil. The level of apoptosis again increased with time following the 1-hour exposure, as illustrated most clearly by the data from the TUNEL assay (Figure 4C).

The results generated using chlorambucil show that induction of apoptosis is drug concentration-dependent, and that significant levels of apoptosis are associated with total growth inhibition. Furthermore, both the time course and level of apoptosis induced by chlorambucil were very similar to those produced by equitoxic concentrations of ZD2767P + CPG2.

*© 2001 Cancer Research Campaign*

BJOC 01-1947 764-771 20/8/01 3:14 pm Page 768

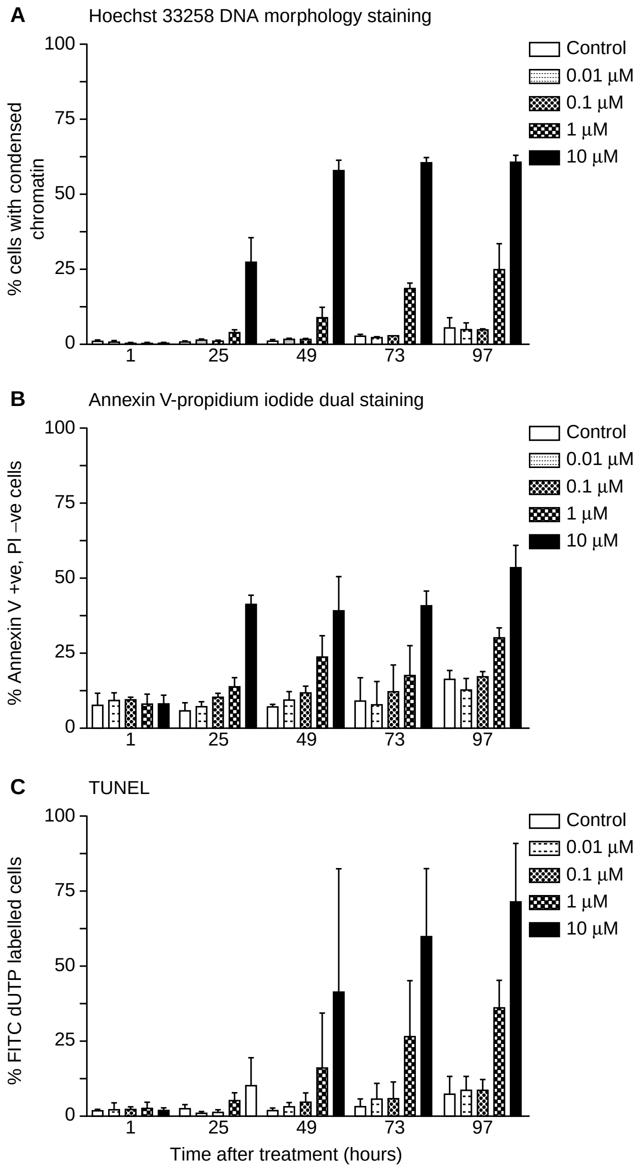

**Figure 3** Time course of apoptosis induction in LoVo colorectal tumour cells after a 1-h exposure to ZD2767P + CPG2. Apoptosis was determined after a 1-h exposure to 0.01, 0.1, 1 or 10 µM ZD2767P + 1 unit/ml CPG2 using (**A**) Hoechst 33258 DNA morphology staining, (**B**) Annexin V-propidium iodide dual staining and (**C**) TUNEL. Each bar represents the mean ± s.d. of *n* = 3 experiments performed as described in Materials and Methods

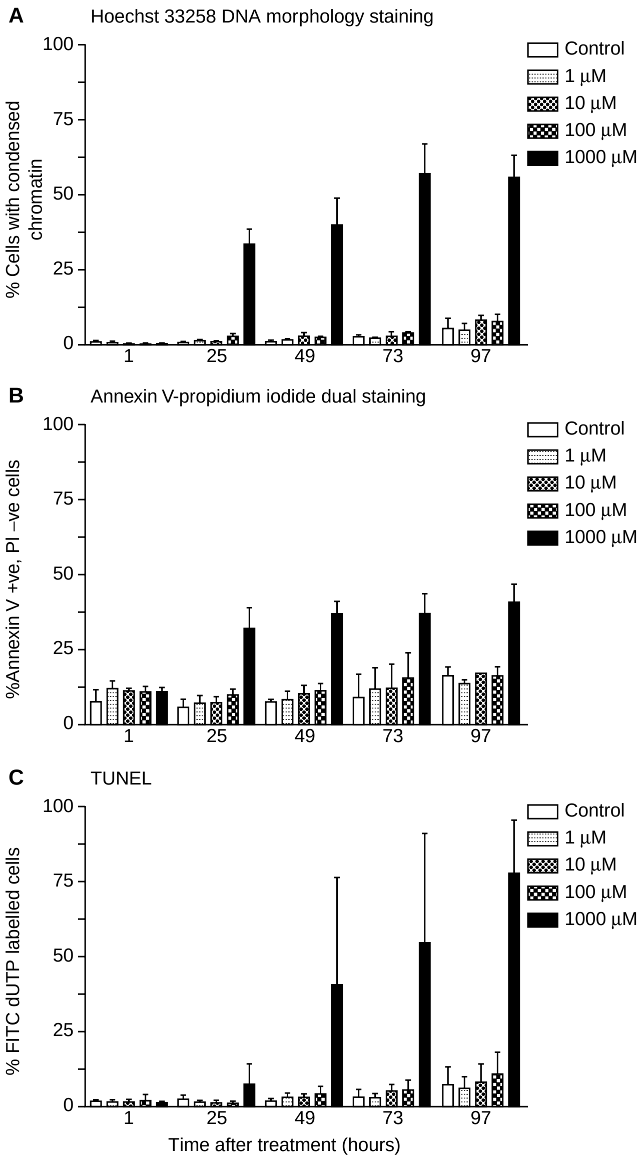

**Figure 4** Time course of apoptosis induction in LoVo colorectal tumour cells after a 1-h exposure to chlorambucil. Apoptosis was determined after a 1-h exposure to 1, 10, 100 or 1000 µM chlorambucil using (**A**) Hoechst 33258 DNA morphology staining, (**B**) Annexin V-propidium iodide dual staining and (**C**) TUNEL. Each bar represents the mean ± s.d. of *n* = 3 experiments performed as described in Materials and Methods

### Induction of apoptosis by the monofunctional ZD2767D analogue

Previously, it has been shown that the level and pattern of sensitivity of a panel colorectal tumour cell lines to the monofunctional ZD2767D analogue is distinct from that of ZD2767P + CPG2 or chlorambucil (Monks et al, submitted). Furthermore, the monofunctional ZD2767D analogue produced different DNA lesions, primarily DNA strand breaks, compared to the bifunctional agents ZD2767P + CPG2 and chlorambucil, which produced DNA-DNA interstrand cross-links. To establish whether the differences in the cellular effects of the monofunctional and bifunctional drugs extended to the mechanism of cell death, the level and time course of apoptosis induced by the monofunctional ZD2767D analogue was determined and compared to data for ZD2767P + CPG2 and chlorambucil.

As observed with the lower concentrations of the bifunctional agents, no apoptosis or growth inhibition was detected at the 1 or 10 µM monofunctional ZD2767D analogue (Figure 5 and Figure 2C, respectively). However, both the Hoechst 33258 DNA morphology staining and Annexin V-PI dual staining assays demonstrated that 100 µM monofunctional ZD2767D analogue induced apoptosis, with maximum levels being reached by 25 h (16% and 22 %, respectively). In contrast, using the TUNEL assay, maximum levels of apoptosis (18%) were not reached until 49 h after treatment with 100 µM of the monofunctional ZD2767D analogue. Unlike the bifunctional alkylating compounds, where levels of apoptosis increased with time, the level of monofunctional ZD2767D analogue-induced apoptosis peaked in all three assays at 25 or 49 h and remained stable for the remainder of the experiment.

 © 2001 Cancer Research Campaign

BJOC 01-1947 764-771 20/8/01 3:14 pm Page 769

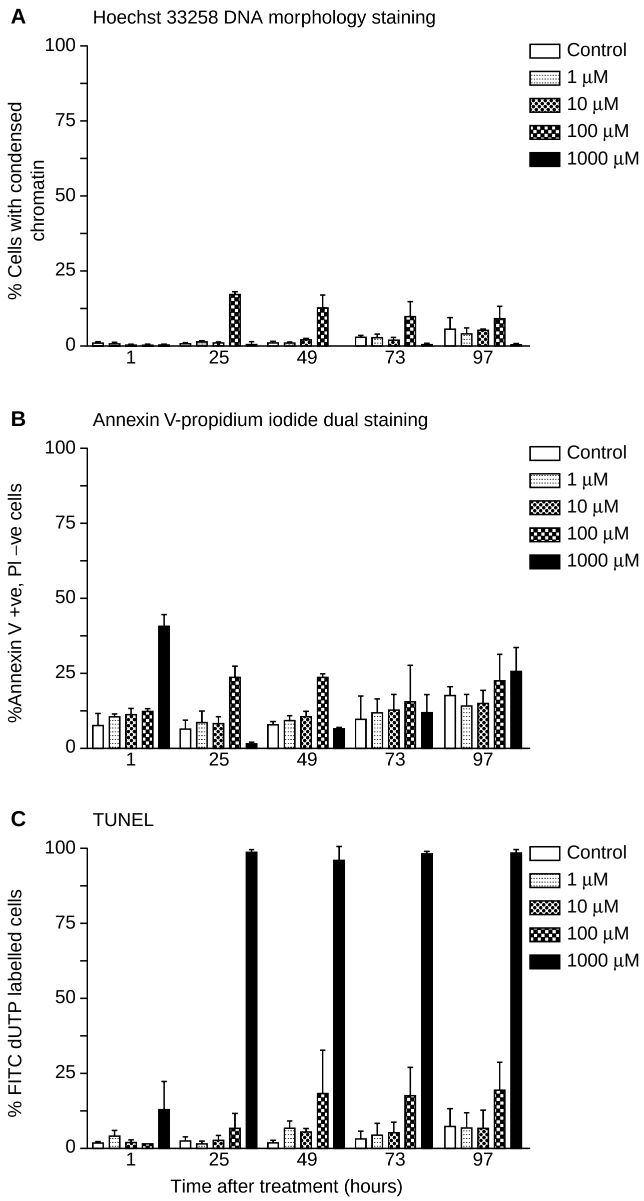

**Figure 5** Time course of apoptosis induction in LoVo colorectal tumour cells after a 1-h exposure to the monofunctional ZD2767D analogue. Apoptosis was determined after a 1-h exposure to 1, 10, 100 or 1000 µM mono-functional ZD2767 analogue using (**A**) Hoechst 33258 DNA morphology staining, (**B**) Annexin V-propidium iodide dual staining and (**C**) TUNEL. Each bar represents the mean ± s.d. of *n* = 3 experiments performed as described in Materials and Methods

Qualitative and quantitative differences in the effects of 1000 µM monofunctional ZD2767D analogue on cells, compared to lower concentrations, were also detected in each of the apoptosis assays. Firstly, no chromatin condensation was seen at any of the five time points studied following 1000 µM monofunctional ZD2767D analogue treatment as analysed by the Hoechst 33258 DNA morphology assay (Figure 5A). Secondly, the Annexin V-PI assay demonstrated high levels (39%) of An V +ve / PI –ve staining at 1 hour suggesting rapid induction of membrane asymmetry. However, by 25 h the level of An V +ve / PI –ve staining had decreased to 2%, a level which was below that in control cells (6%), following which the Annexin V staining increased in line with that seen in the control cells. Thirdly, the TUNEL assay also showed detectable levels of DNA labeling (13%) at 1 h, and by 25 h the number of cells considered to be above the apoptotic threshold was 100%.

The results for the induction of apoptosis by the 1000 µM monofunctional ZD2767D analogue varied depending on the apoptosis assay. When visualized under a light microscope, the cells were found to have decreased in size and to be firmly fixed to the plastic of the tissue culture flask. Removal of the cells was not facilitated by trypsinization but required mechanical force, suggesting that the cells had undergone drug-induced membrane changes which caused a fixation-like attachment to the tissue culture flask.

Following treatment with 1000 µM monofunctional ZD2767D analogue Hoechst 33258 DNA morphology staining showed no chromatin condensation at any of the time points measured, and a level lower than in control cells at 49, 73 and 97 h (Figure 5A). Visualization of the 1000 µM monofunctional ZD2767D analogue treated cells showed the nuclei to be smaller than control nuclei with a homogenous staining pattern and no intra-nuclear structure. In the annexin V-PI assay the cells were largely detected as being apoptotic or late apoptotic/necrotic 1 h after treatment, but by 25 h there was a reduction in the number of apoptotic cells to below control levels (Figure 5B). Analysis of the cell population from 25 h onwards demonstrated that all the cells were late apoptotic/necrotic (Figure 5B). The TUNEL assay also demonstrated increased labeling of DNA with FITC-dUTP at 1 h, and by 25 h after treatment with 1000 µM monofunctional ZD2767D analogue the TUNEL assay showed 100% of the cells to be positively labelled for apoptosis (Figure 5C). Furthermore, the level of FITC-dUTP incorporated into each cell was far above that seen with lower drug concentrations, or with ZD2767P + CPG2 or chlorambucil, suggesting a massive amount of DNA fragmentation in the cells treated with the monofunctional drug.

### DISCUSSION

As part of the investigations into the mechanisms and determinants of ZD2767 ADEPT-induced cytotoxicity, the mode and kinetics of cell death were studied to establish whether ZD2767P + CPG2 produces cell death via similar mechanisms to those of established nitrogen mustards such as chlorambucil.

To investigate the mechanism of cell death induced by ZD2767P + CPG2, three different apoptosis assays were used, each assay measuring apoptosis by assessing a distinct and different morphological characteristic. Chromatin condensation was visualised using the Hoechst 33258 DNA binding stain, loss of membrane phospholipid asymmetry and selective permeability were studied using the flow cytometric annexin V-propidium iodide assay, and measurement of inter-nucleosomal DNA cleavage fragments by the flow cytometric TUNEL assay.

Exposure of LoVo colorectal tumour cells to ZD2767P + CPG2 for 1 h resulted in a concentration-dependent increase in the level of apoptosis. All three assays demonstrated the induction of similar levels of apoptosis which only occurred at ZD2767P + CPG2 concentrations that induced of growth inhibition (1 and 10 µM). The level of apoptosis was found to increase with respect to time at both the 1 and 10 µM ZD2767P + CPG2 concentrations in all three assays.

To establish whether the level and time course of apoptosis induced by ZD2767P + CPG2 was similar to that produced

*© 2001 Cancer Research Campaign*

by classical nitrogen mustards, chlorambucil was used as a comparator. A 1 h exposure to chlorambucil also resulted in a time-and concentration-dependent increase in the level of apoptosis in LoVo colorectal tumour cells. Only 1000 μM chlorambucil showed an increase in the level of apoptosis above that in untreated cells, consistent with the observation that it was only this concentration that inhibited cell growth. One thousand μM chlorambucil produced complete growth inhibition, as did 10 μM ZD2767P + CPG2, and the 2 treatments resulted in similar levels of apoptosis at each time point in all three assays.

Time and dose-dependency of apoptosis has been demonstrated previously using the annexin V-propidium iodide dual-staining assay in the human leukaemia cell line HSB2, following exposure to the anti-cancer agents cladribine, cytarabine, cisplatin and 5-fluorouracil (Guchelaar et al, 1998), and in peripheral blood B-lineage chronic lymphocytic leukaemia cells treated with chlorambucil (Pepper et al, 1998a). Furthermore, as observed in the current study, the level of chlorambucil-induced apoptosis in B-lineage chronic lymphocytic leukaemia cells, measured using the annexin V-propidium iodide method, was confirmed by, and correlated closely with, the levels detected by the TUNEL assay (Pepper et al, 1998b).

To establish whether the chemical bifunctionality of ZD2767P + CPG2 influences the mode of cell death and kinetics of apoptosis, LoVo colorectal tumour cells were treated with the monofunctional ZD2767D analogue for 1 h. As observed with both ZD2767P + CPG2 and chlorambucil, the induction of apoptosis by the monofunctional ZD2767D analogue was concentration-dependent. Apoptosis, above the levels seen in control cells, was only observed following treatment with 100 μM monofunctional ZD2767D analogue, a concentration which induced growth inhibition. However, in contrast to the results obtained with the bifunctional agents, the level of apoptosis induced by 100 μM monofunctional ZD2767D analogue did not increase with time. Indeed, the level of apoptosis at 100 μM remained constant after 25–49 h in all three assays (16–23%), and did not increase to the levels seen following ZD2767P + CPG2 or chlorambucil exposure (50–70%).

The differences in the levels and time-course of apoptosis-induction by the monofunctional ZD2767D analogue are probably a direct result of the different type of damage induced by a monofunctional versus a bifunctional agent. As described previously (Monks et al, submitted), the monofunctional ZD2767D analogue leads to the formation of high levels of DNA strand breaks, rather than the DNA cross-links seen with the bifunctional alkylating agents ZD2767P + CPG2 and chlorambucil, which presumably represent different apoptotic signals.

An explanation for the differences in the time course and level of apoptosis induced by the mono-functional ZD2767D analogue, compared to the bifunctional agents ZD2767P + CPG2 and chlorambucil, may be that DNA-DNA interstrand cross-links are only detected during DNA replication, by inhibiting strand separation at the replication fork, whilst strand breaks could be detected and induce apoptosis in all phases of the cell cycle. Comparisons of the survival and DNA synthesis delay of HeLa cells exposed to mustard gas and half mustard gas, demonstrated that both compounds produced dose-dependent DNA synthesis inhibition, although a higher concentration of the half mustard was needed to produce a similar effect to the bi-functional mustard (Roberts et al, 1971).

Similarities were observed between the characteristics of cell death induced by 1000 μM mono-functional ZD2767D analogue and necrosis. Consistent with the data for the mon-functional drug, necrosis does not induce chromatin condensation; however, there is rapid loss of selective membrane permeability and extensive nuclear degradation due to the activation of hydrolytic nucleases and proteases which result in the formation of mono-and oligonucleotides (Umansky, 1996). Consistent with necrotic cell death, 1000 μM monofunctional ZD2767D analogue induced rapid loss of selective membrane permeability and extensive levels of early FITC-dUTP staining. A possible explanation for the extensive FITC-dUTP labelling in the TUNEL assay is that high levels of DNA degradation occurred, either due to the extensive non-specific (random) DNA cleavage which occurs during necrosis (Fernandes and Cotter, 1994; Lennon et al, 1991) or to the high levels of DNA strand breaks produced directly by the mono-functional ZD2767D analogue (Monks et al, submitted). Either of these processes could have resulted in the high levels of FITC-dUTP labelling seen in the TUNEL assay. Differences in the mode of cell death has previously been demonstrated in HL60 cells exposed to numerous anti-cancer agents, including chlorambucil (Lennon et al, 1991), where it was shown that low drug concentrations induced apoptosis (detected by chromatin condensation), whilst higher levels induced necrosis (shown by loss of membrane integrity). These latter results demonstrate that an alkylating agent can induce cell death by different modes and are consistent with the hypothesis that at 1000 μM the monofunctional ZD2767D analogue induced cell death by necrosis, or a mixture of necrosis and apoptosis, whereas apoptosis was the predominant mode of cell death at 100 μM. In the context of clinical studies with ZD2767 ADEPT, given the potency of ZD2767P + CPG2, it is extremely unlikely that concentrations of monofunctional alkylating agents will be generated that are able to induce necrosis, and apoptosis is likely to be the sole mechanism of cell killing in patients.

In summary, the studies presented here have demonstrated that ZD2767P + CPG2 induces apoptosis in a time and concentration dependent manner, similar to that of the classical bifunctional alkylating agent chlorambucil. Apoptosis only exceeded levels in control cells at drug concentrations which produced growth inhibition, with cytostatic concentrations of chlorambucil and ZD2767P + CPG2 giving rise to similar levels of apoptosis. Time and concentration dependent induction of apoptosis was also seen following exposure to growth inhibitory concentrations of the monofunctional ZD2767D analogue, although the level and time course of induction was different to that produced by the bifunctional agents. The differences between the kinetics of apoptosis and the modes of cell death induced by the bifunctional alkylating agents (ZD2767P + CPG2 and chlorambucil) and the monofunctional ZD2767D analogue, are consistent with the different types of DNA lesion produced by the agents.

### ACKNOWLEDGEMENTS

This study was supported by grants from the Cancer Research Campaign and AstraZeneca Pharmaceuticals.

### REFERENCES

Bagshawe KD (1987) Antibody directed enzymes revive anti-cancer prodrugs concept. *Br J Cancer* **56**: 531–532

Begleiter A, Lee K, Israels LG, Mowat MRA and Johnston JB (1994) Chlorambucil induced apoptosis in chronic lymphocytic leukemia (CLL) and it’s relationship to clinical efficacy. *Leukemia* **8** (Supp. 1): S103–S106

 *© 2001 Cancer Research Campaign*

BJOC 01-1947 764-771 20/8/01 3:14 pm Page 771

Blakey DC, Burke PJ, Davies DH, Dowell RI, East SJ, Eckersley KP, Fitton JE, McDaid J, Melton RG, Niculescu-Duvaz IA, Pinder PE, Sharma SK, Wright AF and Springer CJ (1996) ZD2767, an improved system for antibody-directed enzyme prodrug therapy that results in tumour regressions in colorectal tumour xenografts. *Cancer Res* **56**: 3287–3292

Chen T (1977) In Situ detection of mycoplasma contamination in cell cultures by fluorescent Hoechst 33258 stain. *Exp Cell Res* **104**: 255–262

Fadok VA, Voelker DR, Campbell PA, Cohen JJ, Bratton DL and Henson PM (1992) Exposure of phosphatidylserine on the surface of apoptotic lymphocytes triggers specific recognition and removal by macrophages. *J Immunol* **148**: 2207–2216

Fernandes RS and Cotter TG (1994) Apoptosis or necrosis: Intracellular levels of glutathione influence mode of cell death. *Biochem Pharmacol* **48**: 675–681

Gavrieli Y, Sherman Y and Ben-Sasson SA (1992) Identification of programmed cell death in situ via specific labeling of nuclear DNA fragmentation. *J Cell Biol* **119**: 493–501

Guchelaar H-J, Vermes I, Koopmans RP, Reutelingsperger CPM and Haanen C (1998) Apoptosis-and necrosis-inducing potential of cladribine, cytarabine, cisplatin and 5-fluorouracil in vitro: a quantitative pharmacodynamic model. *Cancer Chemother Pharmacol* **42**: 77–83

Hickman JA and Boyle CC (1997) Apoptosis and Cytotoxins. *Br Med Bull* **52**: 632–643

Lennon SV, Martin SJ and Cotter TG (1991) Dose-dependent induction of apoptosis in human tumour cell lines by widely diverging stimuli. *Cell Prolif* **24**: 203–214

Lootiens FG Regenfuss P, Zechel A, Dumortier L and Clegg RM (1990) Binding characteristics of Hoechst 33258 with calf thymus DNA, poly[d(A-T)], and d(CCGGAATTCCGG): Multiple stoichiometries and determination of tight binding with a wide spectrum of site affinities. *Biochemistry* **29**: 9029–9039

Martin SJ, Reutelingsperger CPM, McGahon AJ, Rader JA, van Schie RCAA, LaFace DM and Green DR (1995) Early redistribution of plasma membrane phosphatidylserine is a general feature of apoptosis regardless of the initiating stimulus: Inhibition by overexpression of Bcl-2 and Abl. *J Exp Med* **182**: 1545–1556

Pepper C, Hoy T and Bentley P (1997) Bcl-2/Bax ratios in chronic lymphocytic leukaemia and their correlation with *in vitro* apoptosis and clinical resistance. *Br J Cancer* **76**: 935–938

Pepper C, Hoy T and Bentley P (1998a) Elevated Bcl-2/Bax are a consistent feature of apoptosis resistance in Bcl-2 chronic lymphocytic leukaemia and are correlated with *in vivo* chemoresistance. *Leukemia and Lymphoma* **28**: 355–360

Pepper C, Thomas A, Tucker H, Hoy T and Bentley P (1998b) Flow cytometric assessment of three different methods for the measurement of in vitro apoptosis. *Leuk Res* **22**: 439–444

Roberts JJ, Brent TP and Crathorn AR (1971) Evidence for the inactivation and repair of the mammalian DNA template after alkylation by mustard gas and half mustard gas. *Eur J Cancer* **7**: 515–524

Sgonc R, Boeck G, Dietrich H, Gruber J, Recheis H and Wick G (1994) Simultaneous determination of cell surface antigens and apoptosis. *Trends Genet* **10**: 41–42

Sherwood RF, Melton RG, Alwan SM and Hughes P (1985) Purification and properties of carboxypeptidase G2 from *Pseudomonas* sp. strain RS-16. Use of a novel triazine dye affinity method. *Eur J Biochem* **148**: 447–453

Springer CJ, Dowell R, Burke PJ, Hadley E, Davies DH, Blakey DC, Melton RG and Niculescu-Duvaz I (1995) Optimization of alkylating agent prodrugs derived from phenol and aniline mustards: A new clinical candidateprodrug (ZD2767) for antibody-directed enzyme prodrug therapy (ADEPT). *J Med Chem* **38**: 5051–5065

Umansky SR (1996) Apoptosis: Molecular and cellular mechanisms (A review). *Mol Biol* **30**: 285–295

Vermes I, Haanen C, Steffens-Nakken H and Reutelingsperger C (1995) A novel assay for apoptosis flow cytometric detection of phosphatidylserine expression on early apoptotic cells using fluorescein labelled annexin V. *J Immunol Methods* **184**: 39–51

*© 2001 Cancer Research Campaign*

## References

[PDF_00795] Bagshawe K. D. (1987). Antibody directed enzymes revive anti-cancer prodrugs concept.. Br J Cancer.

[PDF_00797] Begleiter A., Lee K., Israels L. G., Mowat M. R., Johnston J. B. (1994). Chlorambucil induced apoptosis in chronic lymphocytic leukemia (CLL) and its relationship to clinical efficacy.. Leukemia.

[PDF_00803] Blakey D. C., Burke P. J., Davies D. H., Dowell R. I., East S. J., Eckersley K. P., Fitton J. E., McDaid J., Melton R. G., Niculescu-Duvaz I. A. (1996). ZD2767, an improved system for antibody-directed enzyme prodrug therapy that results in tumor regressions in colorectal tumor xenografts.. Cancer Res.

[PDF_00808] Chen T. R. (1977). In situ detection of mycoplasma contamination in cell cultures by fluorescent Hoechst 33258 stain.. Exp Cell Res.

[PDF_00810] Fadok V. A., Voelker D. R., Campbell P. A., Cohen J. J., Bratton D. L., Henson P. M. (1992). Exposure of phosphatidylserine on the surface of apoptotic lymphocytes triggers specific recognition and removal by macrophages.. J Immunol.

[PDF_00814] Fernandes R. S., Cotter T. G. (1994). Apoptosis or necrosis: intracellular levels of glutathione influence mode of cell death.. Biochem Pharmacol.

[PDF_00816] Gavrieli Y., Sherman Y., Ben-Sasson S. A. (1992). Identification of programmed cell death in situ via specific labeling of nuclear DNA fragmentation.. J Cell Biol.

[PDF_00819] Guchelaar H. J., Vermes I., Koopmans R. P., Reutelingsperger C. P., Haanen C. (1998). Apoptosis- and necrosis-inducing potential of cladribine, cytarabine, cisplatin, and 5-fluorouracil in vitro: a quantitative pharmacodynamic model.. Cancer Chemother Pharmacol.

[PDF_00823] Hickman J. A., Boyle C. C. (1997). Apoptosis and cytotoxins.. Br Med Bull.

[PDF_00825] Lennon S. V., Martin S. J., Cotter T. G. (1991). Dose-dependent induction of apoptosis in human tumour cell lines by widely diverging stimuli.. Cell Prolif.

[PDF_00828] Loontiens F. G., Regenfuss P., Zechel A., Dumortier L., Clegg R. M. (1990). Binding characteristics of Hoechst 33258 with calf thymus DNA, poly[d(A-T)], and d(CCGGAATTCCGG): multiple stoichiometries and determination of tight binding with a wide spectrum of site affinities.. Biochemistry.

[PDF_00833] Martin S. J., Reutelingsperger C. P., McGahon A. J., Rader J. A., van Schie R. C., LaFace D. M., Green D. R. (1995). Early redistribution of plasma membrane phosphatidylserine is a general feature of apoptosis regardless of the initiating stimulus: inhibition by overexpression of Bcl-2 and Abl.. J Exp Med.

[PDF_00838] Pepper C., Hoy T., Bentley D. P. (1997). Bcl-2/Bax ratios in chronic lymphocytic leukaemia and their correlation with in vitro apoptosis and clinical resistance.. Br J Cancer.

[PDF_00841] Pepper C., Hoy T., Bentley P. (1998). Elevated Bcl-2/Bax are a consistent feature of apoptosis resistance in B-cell chronic lymphocytic leukaemia and are correlated with in vivo chemoresistance.. Leuk Lymphoma.

[PDF_00845] Pepper C., Thomas A., Tucker H., Hoy T., Bentley P. (1998). Flow cytometric assessment of three different methods for the measurement of in vitro apoptosis.. Leuk Res.

[PDF_00848] Roberts J. J., Brent T. P., Crathorn A. R. (1971). Evidence for the inactivation and repair of the mammalian DNA template after alkylation by mustard gas and half mustard gas.. Eur J Cancer.

[PDF_00851] Sgonc R., Boeck G., Dietrich H., Gruber J., Recheis H., Wick G. (1994). Simultaneous determination of cell surface antigens and apoptosis.. Trends Genet.

[PDF_00854] Sherwood R. F., Melton R. G., Alwan S. M., Hughes P. (1985). Purification and properties of carboxypeptidase G2 from Pseudomonas sp. strain RS-16. Use of a novel triazine dye affinity method.. Eur J Biochem.

[PDF_00857] Springer C. J., Dowell R., Burke P. J., Hadley E., Davis D. H., Blakey D. C., Melton R. G., Niculescu-Duvaz I. (1995). Optimization of alkylating agent prodrugs derived from phenol and aniline mustards: a new clinical candidate prodrug (ZD2767) for antibody-directed enzyme prodrug therapy (ADEPT).. J Med Chem.

[PDF_00864] Vermes I., Haanen C., Steffens-Nakken H., Reutelingsperger C. (1995). A novel assay for apoptosis. Flow cytometric detection of phosphatidylserine expression on early apoptotic cells using fluorescein labelled Annexin V.. J Immunol Methods.

